# Comparison of intestinal bacterial and fungal communities across various xylophagous beetle larvae (Coleoptera: Cerambycidae)

**DOI:** 10.1038/s41598-018-27342-z

**Published:** 2018-07-03

**Authors:** Waleed S. Mohammed, Elvira E. Ziganshina, Elena I. Shagimardanova, Natalia E. Gogoleva, Ayrat M. Ziganshin

**Affiliations:** 1 Department of Microbiology, Institute of Fundamental Medicine and Biology, Kazan (Volga Region) Federal University, Kazan, 420008 Russia; 2 Department of Biotechnology, Faculty of Agriculture, Al-Azhar University, Cairo, 11651 Egypt; 3 Laboratory of Extreme Biology, Institute of Fundamental Medicine and Biology, Kazan (Volga Region) Federal University, Kazan, 420021 Russia

## Abstract

The microbial gut communities associated with various xylophagous beetles offer great potential for different biotechnologies and elaboration of novel pest management strategies. In this research, the intestinal bacterial and fungal communities of various cerambycid larvae, including *Acmaeops septentrionis*, *Acanthocinus aedilis*, *Callidium coriaceum*, *Trichoferus campestris* and *Chlorophorus herbstii*, were investigated. The intestinal microbial communities of these Cerambycidae species were mostly represented by members of the bacterial phyla Proteobacteria and Actinobacteria and the fungal phylum Ascomycota. However, the bacterial and fungal communities varied by beetle species and between individual organisms. Furthermore, bacterial communities’ metagenomes reconstruction indicated the genes that encode enzymes involved in the lignocellulose degradation (such as peroxidases, alpha-L-fucosidases, beta-xylosidases, beta-mannosidases, endoglucanases, beta-glucosidases and others) and nitrogen fixation (nitrogenases). Most of the predicted genes potentially related to lignocellulose degradation were enriched in the *T. campestris,* *A. aedilis* and *A. septentrionis* larval gut consortia, whereas predicted genes affiliated with the nitrogenase component proteins were enriched in the *T. campestris*, *A. septentrionis* and *C. herbstii* larval gut consortia. Several bacteria and fungi detected in the current work could be involved in the nutrition of beetle larvae.

## Introduction

Insecta is the most diverse class of animals, living in multiple habitats and feeding on various substrates^1^. Insects are colonized by different microorganisms, which are often beneficial or required by the hosts. Also, the intestinal systems of insects are excellent places for the growth of a wide variety of microorganisms^2–4^.

Cerambycidae (longhorned beetles or longicorns) is one of the largest families of beetles^2^. All cerambycids are plant feeders, and most cerambycid larvae feed on healthy, dead, or decaying solid plant tissues or, less often, rotten softwoods. Cerambycid beetles can damage forest trees and dry structural wood timbers^2,5^. Xylophagous beetles, which belong to one of the most important groups of arthropods, produce the enzymes necessary to digest many components of woody material by themselves; alternatively, these components can be degraded by the activity of xylophagous beetles’ intestinal microbial communities^3,6^. While the anatomy and physiology of many Cerambycidae species are well understood, little is known about the complex bacterial and fungal consortia associated with their gut systems.

The symbiotic relationships between prokaryotes/fungi and insects are one of the principal research areas of microbial ecology. One of the most promising examples of such symbiotic relationships is between xylophagous insects and their intestinal communities. According to previous research^4,7^, symbiotic microbes can live in a variety of insect species, such as beetles, termites, cockroaches and others. The diet of the cerambycid larvae is poor in available nutrients but rich in fibers, which are hard to digest^6^. It was also reported that intestinal microorganisms can convert lignin in the gut systems of some cerambycid beetles^8^. Several bacterial species isolated from beetle guts can additionally metabolize terpenoid molecules, which helps the beetles to colonize the host trees^9^. In addition, low levels of amino-nitrogen are present in bark and wood; therefore, many xylophagous insects have stable associates that may help them colonize such exceptional ecological niches.

Investigating the intestinal bacterial and fungal consortia structures of xylophagous insects with limited diets is necessary to better understand the potential role of gut microbes in cellulose/hemicellulose/lignin transformation, nitrogen fixation and other processes. Also, the intestinal microbes can protect the hosts against pathogens and parasites. Moreover, microorganisms within the insect intestine are important sources of novel enzymes and can be used in biological control and new pest management strategies^7^.

Next-generation sequencing technologies cost-effectively generate enormous amounts of sequencing data and enable researchers across biological disciplines to address different research questions^10–14^. Thus, 16S rRNA gene is the commonly used phylogenetic marker to analyze the prokaryotic communities structure in various environments^2,4,14–16^. The internal transcribed spacer (ITS) region is the most recommended as the universal DNA barcode marker to identify fungi^17^. These classical markers are useful to investigate the communities structure but do not give information on the genes involved in the lignocellulose transformation and nitrogen fixation. Recently, Langille *et al*.^18^ developed the PICRUSt software to predict the functional composition of metagenomes by using marker gene data and a database of reference genomes.

In this study, larvae of five species of cerambycid beetles, such as *Acmaeops septentrionis*, *Acanthocinus aedilis*, *Callidium coriaceum*, *Trichoferus campestris* and *Chlorophorus herbstii*, were investigated. We intended to receive a detailed overview of the intestinal bacterial and fungal communities associated with these wood-inhabiting cerambycid larvae and to get new knowledge about the potential impacts of microbes on their beetle hosts. Despite the ecological and agricultural interest of these beetles species, knowledge of the composition and diversity of bacterial and fungal communities associated with their gut systems is lacking.

## Results

### Identification of species of beetles

Since the identification of many beetle species and especially of larval stages using only morphological data can be complicated and time-consuming^5,19^, in the present study, in addition to the morphological methods, we analyzed the mitochondrial cytochrome *c* oxidase I (COI) gene for accurate and reliable differentiation and identification of larvae of different bark- and wood-inhabiting cerambycid beetles that inhabit the Republic of Tatarstan (Russian Federation). Additionally, we compared the obtained COI gene dataset with the 18S rRNA gene dataset (V7 region).

We investigated thirty-five specimens representing five species and five genera of cerambycid beetles at the larval stage of their development. The mitochondrial COI gene and the V7 region were successfully PCR amplified and sequenced, confirming the universality of the used primers for these cerambycid beetles. There was no indication of nuclear mitochondrial pseudogenes (numts) amplification in PCR reactions for the received COI dataset. The overall mean K2P distance and *p*-distance for the COI dataset were 19.8% and 17.1%, respectively. The highest observed interspecific K2P distance was 26.0% (*p*-distance: 21.8%) between *T. campestris* (TC) and *A. septentrionis* (AS), while the lowest distances were detected between *A. aedilis* (AA) and *A. septentrionis* (AS) (K2P distances: 18.8%, *p*-distances: 16.5%; Supplementary Table S1). Intraspecific distances ranged from 0 up to 1.7% (*p*-distances: 0.0 to 1.7%) for AS, AA, CC, TC species, while specimens of *C. herbstii* (CH) were characterized by K2P distances ranging up to 2.5% (*p*-distances: 2.4%).

**Figure 1:**
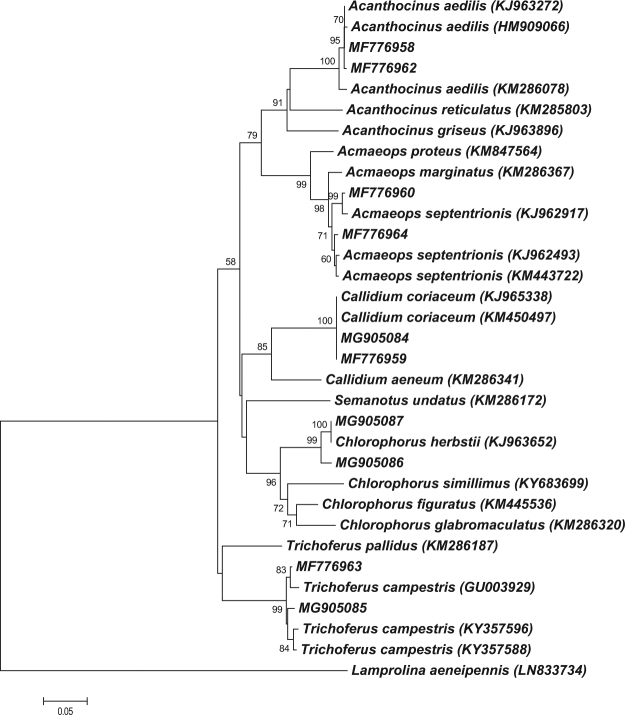
Neighbor-joining tree of COI sequence dataset for cerambycid beetles. The percentage of replicate trees in which the associated taxa clustered together in the bootstrap test (1000 replicates) are shown next to the branches. The tree is drawn to scale, with branch lengths in the same units as those of the evolutionary distances used to infer the phylogenetic tree. The evolutionary distances were computed using the Kimura 2-parameter method and are in the units of the number of base substitutions per site. The analysis involved 33 nucleotide sequences. *Lamprolina aeneipennis* (*Chrysomelidae*) was used as an outgroup taxon.

The neighbor-joining tree prepared from the received COI nucleotide sequences is illustrated in Fig. 1. The used mitochondrial COI gene analysis allowed a better resolution, differentiation and identification of larvae of bark- and wood-inhabiting cerambycid species. In accordance with the COI gene analysis, we could confirm the identity of all five beetle species – AS, AA, CC, TC and CH. Furthermore, we could separate all five species using the V7 marker, but could not identify them as the reference sequences for these cerambycid species were absent in the NCBI database. Therefore, 18S rRNA gene can be applied as an additional molecular marker gene to the COI barcode region for the investigated cerambycid beetles. Finally, the received data show that COI gene represents the most helpful and effective molecular marker for the selected cerambycid species differentiation and identification.

### Bacterial communities associated with the Cerambycidae larval guts

A total of 3,595,297 high-quality-filtered sequences were assigned to 146 operational taxonomic units (OTUs) after processing of all samples. Summary statistics for the Illumina MiSeq runs for all samples are shown in Supplementary Table S2. In general, the 16S rRNA amplicon sequencing sufficiently covered most of the bacterial phylotypes. Comparison of the alpha diversity within the gut microbiome of various larvae was performed on equalized-sequence number (performed by rarefaction; 33,000 reads). Supplementary Table S3 shows alpha diversity indices, such as OTU numbers, Shannon entropy, Simpson, Chao 1, Fisher’s alpha indices, phylogenetic diversity values, while Supplementary Fig. S1 (box plots of observed bacterial OTUs, phylogenetic diversity, Fisher’s alpha and Chao 1 values) illustrates the comparison between the alpha diversity means of each beetle species. In general, the bacterial communities were characterized by higher diversities in gut samples received from *T. campestris* and *C. coriaceum* larvae.

The relative abundance of bacterial groups in the gut content was examined at different taxonomic levels (phylum, family and genus). The classification analysis of the received bacterial reads at phylum level is demonstrated in Supplementary Fig. S2. Members of the phyla Proteobacteria and Actinobacteria were detected in all cerambycids but in different proportions. In total, 12 bacterial phyla and candidate divisions were recovered from all samples. Proteobacteria was found to be the major phylum in most of the CC, TC, CH, AS and AA larval guts. Actinobacteria was additionally found at high levels in several gut samples retrieved from AS and TC, while Firmicutes and/or Cyanobacteria phylotypes were mainly detected in the guts of AA larvae. Interestingly, the candidate phylum OD1 was detected at substantial levels in all CH larvae. Several other phyla accounting over 0.5% of the obtained sequences at least in one of the cerambycid’s gut samples were Acidobacteria, Bacteroidetes, Chlamydiae, Verrucomicrobia, Thermi as well as candidate phyla GN02 and TM7 (Supplementary Fig. S2).

**Figure 2:**
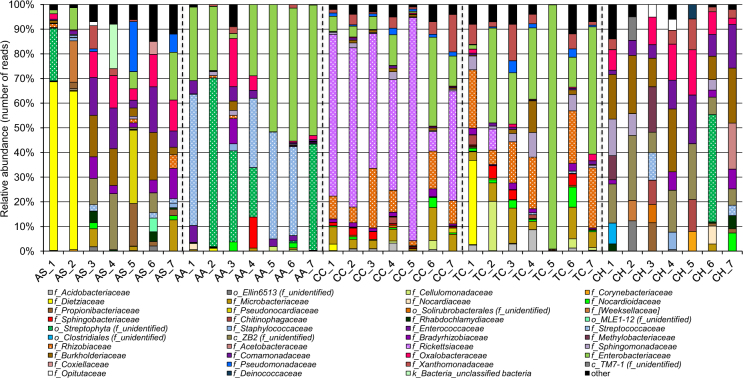
Relative abundance of bacterial families (based on 16S rRNA gene) within cerambycid larval guts. Abbreviations in the figure are given in accordance with the scientific names of beetles (*A. septentrionis* – AS, *A. aedilis* – AA, *C. coriaceum* – CC, *T. campestris* – TC and *C. herbstii* – CH) and the order of the individual larva (1–7). Only families comprising at least 5% relative abundance in at least one sample are shown.

Investigating samples obtained from AS species revealed many taxa affiliated with different families (Fig. 2). Despite a certain overlap, the bacterial communities of *Acmaeops* insects were diverse when comparing the gut content of beetle individuals. Thus, two individuals harbored a high proportion of the family Dietziaceae (Actinobacteria), the next group (four individuals) – Bradyrhizobiaceae, Burkholderiaceae, Comamonadaceae and Oxalobacteraceae (Proteobacteria), while the bacterial community of one *Acmaeops* individual was dominated by the families Pseudonocardiaceae, Propionibacteriaceae (Actinobacteria) and Pseudomonadaceae (Proteobacteria). Other important bacterial families within AS individuals were also observed, but their presence and abundance depended on the investigated larval gut. Various representatives of the Enterobacteriaceae (Proteobacteria) were mainly detected in all AA gut samples; however, the communities of all *Acanthocinus* individuals were also complex and additionally comprised Streptococcaceae (Firmicutes) and unclassified Streptophyta (Cyanobacteria) as well as many other families (but depending on the individual larva). Further analysis demonstrated that Rickettsiaceae, Rhizobiaceae, Enterobacteriaceae (Proteobacteria) and Microbacteriaceae (Actinobacteria) were mainly specific families for all CC larvae, while Enterobacteriaceae, Rhizobiaceae, Xanthomonadaceae (Proteobacteria) and Microbacteriaceae (Actinobacteria) comprised most the bacterial communities in all TC larval guts (but in different proportions). The bacterial communities in the larval guts of CH were dominated by the common candidate class ZB2 and the families Burkholderiaceae, Comamonadaceae and Oxalobacteraceae (Proteobacteria) (Fig. 2). Technical replicates obtained from five samples indicated the good reproducibility of the Illumina sequencing approach (Supplementary Fig. S3).

**Figure 3:**
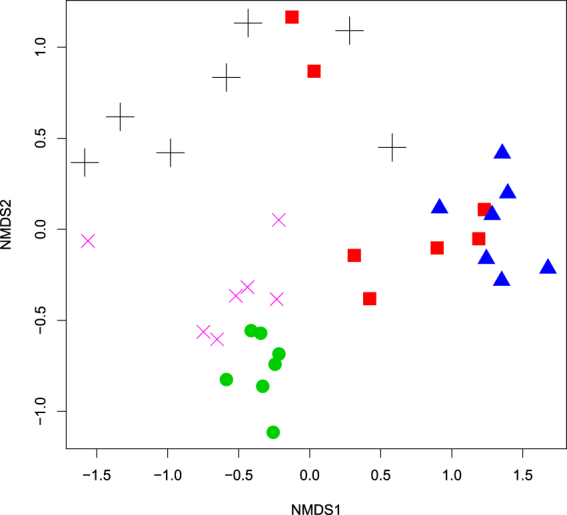
Non-metric multidimensional scaling analysis of the Bray-Curtis dissimilarity index of the bacterial community OTUs (≥97% identity) based on Illumina sequencing of 16S rRNA genes (stress value: 0.14). Symbols: *A. septentrionis* – red square; *A. aedilis* – black cross; *C. coriaceum* – green circle; *T. campestris* – magenta cross; *C. herbstii* – blue triangle.

Supplementary Fig. S4 demonstrates the relative abundances of the most abundant genera observed in specific beetle gut microbiota. We also compared the bacterial community diversity using the NMDS analysis (Fig. 3). Despite different dissimilarities between the bacterial communities of one beetle species, the applied diversity analysis mostly revealed the grouping of samples by xylophagous insect species, though several samples of one beetle species were also close to some samples of the other beetle species. Moreover, samples obtained from AS and AA were relatively scattered within the NMDS plot, indicating a more variability of their bacterial communities.

### Fungal communities associated with larval guts

In total, 1,612,846 high-quality fungal ITS2 reads were obtained after processing of thirty-three samples (in the case of *A. aedilis* fourth sample and *C. herbstii* fifth sample, the received data were removed from the downstream analysis because of the low sequence counts). Finally, sequences were assigned to 74 OTUs, much lower compared to the bacterial data. Summary statistics for the Illumina MiSeq runs for all samples are demonstrated in Supplementary Table S2. In general, the ITS sequencing almost completely covered the fungal taxa detected in all analyzed samples. Supplementary Table S4 illustrates alpha diversity indices (OTU numbers, Shannon entropy, Simpson, Chao 1 and Fisher’s alpha indices) at 2,100 reads, which were selected to analyze the diversity of the fungal communities in the larvae of cerambycids, whereas Supplementary Fig. S5 (box plots of observed fungal OTUs, Fisher’s alpha, Chao 1 and Shannon values) illustrates the comparison between the alpha diversity means of each beetle species. In general, the fungal communities associated with cerambycidae beetle larvae were less diverse than the bacterial communities, and the fungal communities were also found to be more diverse in samples obtained from *T. campestris* and *C. coriaceum*, but less diverse in *C. herbstii* larvae.

The relative abundance of fungal groups was estimated at different taxonomic levels (phylum, family and genus). The classification analysis of the obtained fungal sequences at phylum level is presented in Supplementary Fig. S6. Totally, two fungal phyla (Ascomycota and Basidiomycota) were recovered during this analysis. Ascomycota was found to be the most prevalent phylum in all AS, AA, CC, TC and CH, while Basidiomycota was detected as the second major phylum in the gut samples retrieved from all CC and TC larvae.

**Figure 4:**
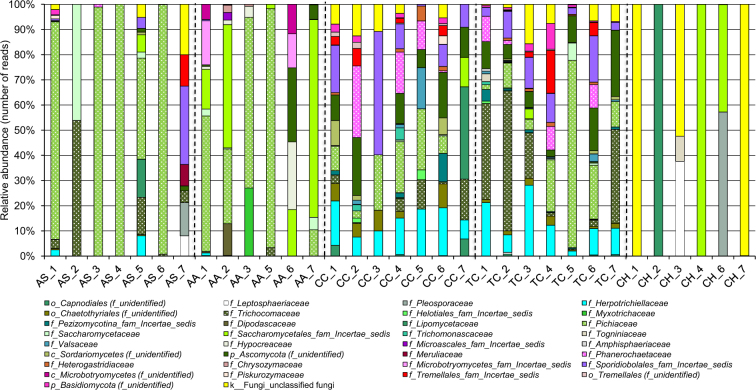
Relative abundance of fungal families (based on ITS sequences) within cerambycid larval guts. Abbreviations in the figure are given in accordance with the scientific names of beetles (*A. septentrionis* – AS, *A. aedilis* – AA, *C. coriaceum* – CC, *T. campestris* – TC and *C. herbstii* – CH) and the order of the individual larva (1–7). AA_4 and CH_5 samples were removed from the analysis due to low sequence numbers.

The fungal communities in the guts of all Cerambycidae species were also variable. During analysis of the fungal OTUs in five gut samples derived from AS, we distinguished major taxa which belonged to the families Pichiaceae (Ascomycota). Moreover, Trichocomaceae and Saccharomycetaceae (Ascomycota) were specific for the second larva, while Sporidiobolales–Family Incertae sedis, Tremellales–Family Incertae sedis, Meruliaceae (Basidiomycota), Pleosporaceae and Leptosphaeriaceae (Ascomycota) were mainly detected in the seventh larva (Fig. 4). Members of the families Pichiaceae and Saccharomycetales–Family Incertae sedis (Ascomycota) were detected in samples retrieved from AA but at different levels (many other families were also observed but their abundances depended on the individual larva). Fungal OTUs detected in CC and TC larvae were very similar and were mainly represented by the common families Herpotrichiellaceae, Pichiaceae, Trichocomaceae and unknown Ascomycota (Ascomycota). In addition, several larvae harbored various basidiomycetes, including representatives of the families Phanerochaetaceae and Sporidiobolales–Family Incertae sedis. In case of CH larvae, the structure of fungal communities was very simple, and almost each CH larva contained specific fungal phylotype(s) with many unidentified fungi (Fig. 4).

**Figure 5:**
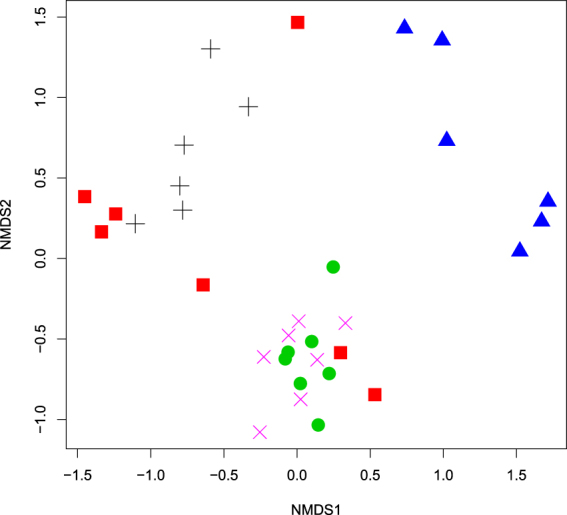
Non-metric multidimensional scaling analysis of the Bray-Curtis dissimilarity index of the fungal community OTUs ( ≥ 97% identity) based on Illumina sequencing of ITS region (stress value: 0.16). Symbols: *A. septentrionis* – red square; *A. aedilis* – black cross; *C. coriaceum* – green circle; *T. campestris* – magenta cross; *C. herbstii* – blue triangle.

Supplementary Fig. S7 illustrates the relative abundances of the abundant fungal genera observed in specific gut microbiota. We also compared the overall fungal communities’ composition by using NMDS analysis (Fig. 5). This analysis mostly demonstrated the clustering of samples by cerambycid beetle species. However, samples obtained from AS were relatively scattered within the NMDS plot, indicating a more variability of their fungal communities, whereas samples of CC were close to samples of TC and some samples of AS.

### Reconstruction of the bacterial communities’ metagenomes using the PICRUSt software

In the present study, metagenome functional profiles of the larval gut bacterial communities were predicted with PICRUSt, a computational approach to predict the functional composition of a metagenome using marker gene datasets and a database of reference genomes^18^. However, this method needs caution in interpreting the received results, because of several factors, including the known impact of horizontal gene transfer between genomes of members of the microbial communities. Additionally, the quality of such functional predictions depends on the availability of annotated reference genomes^18^. The applied approach used here indicated that the most predicted metabolic functional categories were related to metabolism, genetic information and environmental information (Fig. S8). Furthermore, within the metabolism group, the most abundant pathways were related to carbohydrate metabolism, amino acid metabolism, energy metabolism, metabolism of cofactors and vitamins and xenobiotics biodegradation. Cross all pathways groups, predicted genes were highly enriched in the TC larval gut consortia (Fig. S8).

**Figure 6:**
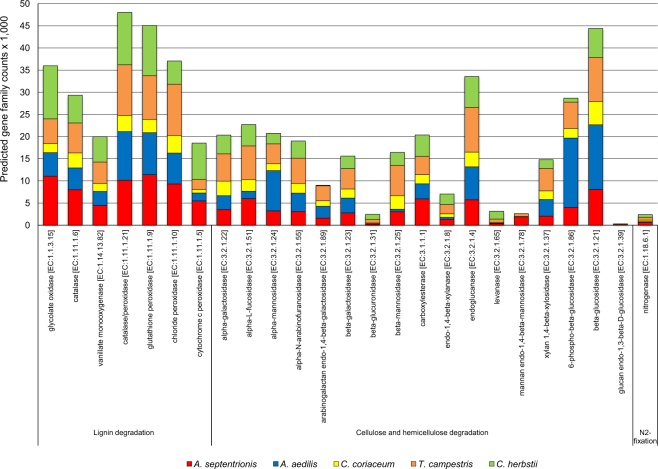
Selection of genes involved in the lignocellulose degradation and nitrogen fixation and the average number of predicted gene family counts (mean values are presented; n = 7). The bacterial communities’ metagenomes were reconstructed using the PICRUSt approach.

Figure 6 shows twenty-four genes potentially related to lignocellulose degradation and the average number of gene family counts predicted by the PICRUSt software^18^, while Supplementary Table S5 demonstrates the enzyme-catalyzed reactions. Thus, when focusing on the lignocellulose deconstruction pathways, genes that codify for catalase/peroxidase (EC:1.11.1.21), glutathione peroxidase (EC:1.11.1.9), chloride peroxidase (EC:1.11.1.10), glycolate oxidase (EC:1.1.3.15), vanillate monooxygenase (EC:1.14.13.82) (related to lignin degradation), endoglucanase (EC:3.2.1.4), alpha-L-fucosidase (EC:3.2.1.51), alpha-mannosidase (EC:3.2.1.24), alpha-N-arabinofuranosidase (EC:3.2.1.55), beta-mannosidase (EC:3.2.1.25), beta-galactosidase (EC:3.2.1.23), xylan 1,4-beta-xylosidase (EC:3.2.1.37) (related to (hemi)cellulose deconstruction) and beta-glucosidase (EC:3.2.1.21) (related to cellobiose decomposition) were predicted; however, the predicted genes abundance depended on the specificity of the beetle species gut environment. Most of the predicted genes potentially related to lignocellulose degradation were enriched in TC, AA and AS larval gut bacterial consortia (Fig. 6). Regarding the biological nitrogen fixation, the key genes for nitrogenase component proteins (*nifH*, *nifD* and *nifK*) were additionally predicted (Fig. 6 and Table S5). Three predicted genes related to nitrogen fixation were enriched in the TC, AS and CH larval gut consortia. A link of phylogeny with functions (such as lignocellulose metabolism and nitrogen fixation) is shown in Supplementary Fig. S9 (with the phylum Proteobacteria with high contributions). The prediction accuracy of PICRUSt in metagenomic larval gut samples was evaluated by quantifying the nearest sequenced taxon index (NSTI), which represents the sum of phylogenetic distances for each organism in the OTU table to its nearest relative with a sequenced reference genome, measured in terms of substitutions per site in the 16S rRNA gene and weighted by the frequency of that organism in the OTU table^18^. NSTI values of most samples were low (0.01–0.08).

## Discussion

The diet of larvae of cerambycid beetles is poor in available nutrients but rich in cellulose, hemicellulose and lignin, which are hard to digest^6^. Also, low levels of amino-nitrogen are present in woody materials^20^. Therefore, investigating the intestinal microbial communities of the wood- and bark-inhabiting insects is important to better understand the potential role of gut microorganisms in contributing to nutrition, lignin/cellulose/hemicellulose degradation, nitrogen fixation and detoxification processes. Also, microbes in the beetles’ intestine are important sources of enzymes for various industries^7^.

The main goal of this work was to characterize the microbial consortia inhabiting the larval gut systems of bark- and wood-inhabiting cerambycid beetles using sequencing of the bacterial 16S rRNA gene and fungal ITS region. Thus, larvae isolated from dead trees or stumps, including *A. septentrionis*, *A. aedilis*, *C. coriaceum*, *T. campestris* and *C. herbstii*, were investigated to find differences between their gut microbial communities. Despite many enzymes necessary for the utilization of organic compounds are produced by the beetle hosts, many important enzymes are also synthesized by transient microorganisms or symbiotic microorganisms associated with the insect gut systems^7^. The molecular approach applied here and received data revealed the high potential of COI barcodes for species differentiation and identification within the family Cerambycidae.

The applied 16S rRNA gene and fungal ITS region sequencing analysis showed that, despite a certain overlap, the bacterial and fungal communities in the intestinal systems of beetle larvae had distinct OTUs differences, most likely due to transient microbes acquired from individual feeding places of larvae (various dead trees) but potentially also due to different stable microbial associations in beetle species. Also, we found a consistent association of bark- and wood-inhabiting cerambycid species mostly with the bacterial phyla Proteobacteria and Actinobacteria and the fungal phylum Ascomycota. The presence and high abundance of members of such bacterial phyla^2,4,21^ and fungal phylum^22,23^ in guts of various other beetle species have been previously reported.

The bacterial communities in the guts of AS larvae were complex and diverse when comparing various beetle individuals. Our characterization of the gut microbiota of AS species by high-throughput sequencing revealed the genera *Dietzia*, *Chryseobacterium*, *Bradyrhizobium*, *Burkholderia*, *Ralstonia*, *Amycolatopsis*, *Pseudomonas*, unknown *Streptophyta*, unclassified ZB2, unclassified *Propionibacteriaceae* and other bacterial groups at notable levels (but their presence and abundances depended on the individual larva). *Dietzia* species are also detected in various environments, including animal intestinal tracts. *Dietzia* strains isolated from the hindgut of the larvae of the Japanese horned beetle *Trypoxylus dichotomus* (Coleoptera: Scarabaeidae) showed the high xylanolytic activity^24^. Bacteria belonging to the genus *Chryseobacterium* can be cellulolytic^25^, and similar bacteria have been previously isolated from many xylophagous insects^26,27^. Carbohydrate fermentation by these and other bacteria identified in the gut content of AS could form a pool of metabolites, which might be used by the host as an energy source. Species of the genus *Bradyrhizobium*^28^, as well as several species of the genera *Ralstonia*, *Burkholderia*^29^ and *Pseudomonas*^30^, isolated from different ecological niches are also capable of nitrogen fixation providing the host with nitrogen. Also, various bacteria might be involved in the detoxification of the secondary plant metabolites and/or protection against pathogens^26^.

Various representatives of the family Enterobacteriaceae were also detected in AA gut samples at notable levels; however, the bacterial communities structure in all AA larvae was also complex, and these communities additionally comprised lactic acid producing *Lactococcus* spp. and unclassified *Streptophyta* (but depending on the individual larva). *Enterobacterales* species have been already found in many other beetles’ guts, where they were implicated in nitrogen-fixation processes and cellulose catabolism^31^, while carbohydrate fermentation by *Lactococcus* bacteria leads to the production of lactic acid which can be used by the host as an energy source^32^. Multifarious members of the *Enterobacterales* were also abundant in xylophagous *Rhagium inquisitor* larvae identified with the 16S rRNA cloning/sequencing techniques^2^. In case of xylophagous CC larvae, bacterial phylotypes observed in their guts were very different when compared to previously described beetle species. Thus, molecular analysis showed that their communities were mostly represented by bacteria belonging to the genera *Wolbachia*, *Agrobacterium*, *Salinibacterium* and *Sodalis*. *Wolbachia* species are most common parasitic microbes and widely distributed among arthropods, including representatives of the Coleoptera, but may belong to mutualistic endosymbionts as well^33,34^. Moreover, representatives of the bacterial genus *Sodalis* have also been described in mutualistic interactions with tsetse flies^35^ and heteropteran insects^36^. Metabolism of members of the genus *Salinibacterium* is respiratory^37^, but organic acids can be produced from several sugars as well, whereas species of the genus *Agrobacterium* can contribute to nitrogen intake by nitrogen-fixing activity^38^.

Several members of the family Enterobacteriaceae, genera *Dietzia*, *Salinibacterium*, *Sphingomonas* and *Luteibacter*, as well as some nitrogen-fixing representatives of the genera *Agrobacterium* and *Rhizobium*, comprised most the bacterial communities in TC larval guts (but depending on the individual larva). *Sphingomonas* and *Luteibacter* bacteria with different metabolic capabilities can destroy the cellulose^39,40^. The predicted genes for lignocellulose degradation and nitrogenase component proteins were also enriched in most of the TC larval guts (Fig. 6), where they might be implicated in lignocellulolytic activities and nitrogen-fixing processes, respectively. Bacterial phylotypes observed in the guts of CH larvae were closely related to the common candidate phylum OD1 (class ZB2) and the order *Burkholderiales*, including *Burkholderia* and *Ralstonia* species, which are also capable of nitrogen fixation^29^, providing the host with nitrogen. *Burkholderia* species have also been described as widespread symbionts of beetles of the genus *Lagria*^41^ and heteropteran insects^42^. Different other bacterial phylotypes were also specific for CH larvae, but their presence and abundance also depended on the investigated individual larva.

In general, less fungal OTUs were detected in the guts of CH larvae and more OTUs were found in the guts of TC and CC larvae. Besides the gut bacterial communities, AS larvae were mostly associated with the Ascomycota, such as representatives of the genera *Hyphopichia*, *Kuraishia*, *Ogataea* (yeasts) and *Aspergillus* (filamentous fungi) as well as several other genera (but their presence and abundance depended on the individual larva). Moreover, one larval gut contained a high level of members of the Basidiomycota, including the yeast *Rhodotorula*. *Hyphopichia* species can be found in various environments; they are also associated with beetles or beetle larval substrates and can ferment different sugars^43^. *Kuraishia* species isolated from diverse wood-associated habitats can ferment various sugars^44^ and were also observed in *Dendroctonus armandi* (*Scolytinae*) gut systems^22^. *Ogataea polymorpha* (as a representative of the genus *Ogataea*) is one of the most important industrially applied yeast, which can ferment xylose and has been studied as a potential producer of ethanol from lignocellulosic biomass^45,46^. *Aspergillus* species can produce a wide range of enzymes important to degrade plant cell wall polysaccharides (such as endoglucanases, exoglucanases, cellobiohydrolases and beta-glucosidases involved in the biodegradation of cellulose; endoxylanases and beta-xylosidases involved in the biodegradation of the xylan backbone; hydrolases and lyases that are the pectin main-chain-degrading enzymes) and can be implicated in lignin conversion as well^47^. Basidiomycota ITS sequences were rarely identified in the samples received from AS larvae except for one larva. Several fungal species were also associated with the guts of AA larvae, such as *Hyphopichia*, *Nakazawaea*, *Candida* (yeasts), *Trichoderma* (filamentous fungi) and many others. *Nakazawaea* species can be found on various plants and substrates associated with them and are probably associated with the bark beetles^48,49^. Symbiotic yeasts related to *Candida* genera (detected in all AA larvae but in different proportions) were also isolated from the digestive tracts, mycetomes of various beetles and can be implicated in different processes^2,50^. *Trichoderma* species comprise several glycoside hydrolases, peroxidases and laccases which are involved in the degradation of lignocellulosic materials^51^.

The composition of fungal communities in CC and TC larvae was very similar between individuals. Thus, within all CC and TC fungal communities, *Exophiala*, *Yamadazyma*, unidentified *Chaetothyriales*, unidentified *Ascomycota* and *Penicillium*-related ITS sequences were distinguished (but at different levels). *Yamadazyma* yeasts can convert lignocellulose and ferment xylose^52^, while *Penicillium* species have an enzymatic machinery to degrade lignocellulosic material, such as lignin peroxidases, beta-exoglucanase, beta-endoglucanase, beta-glucosidase and other enzymes^53,54^. Within the *Basidiomycota*, the yeast *Rhodotorula* was identified in all CC and TC larvae, which can synthesize many important compounds for beetles and have wide industrial applications^55^. In case of CH larvae, their fungal communities displayed very low diversity and varied with several unknown fungi. Many other filamentous fungi and yeasts were detected in the gut content of AS, AA, CC and TC, which were also observed in many other environments (including soil, woody substrates, beetles’ gut systems). They could be involved in the transformation of multifarious types of substrates (carbohydrates, proteins, lipids), providing nutritional needs for the hosts. However, pathogenic effects of some fungi on the hosts cannot be ruled out either. Many wood-feeding insects also have obligate external associations with filamentous fungi and inoculate fungal cells into the food sources, where fungi convert the lignocellulose and serve other nutritional roles^7^. Recently several carrion beetles have been reported to harbor a diversity of ascomycetous yeasts closely related to *Yarrowia lipolytica* (*Yarrowia*-related symbionts)^23,56^, which represents as a major biotechnological interest for production of organic acids and bioremediation of contaminated environments^57,58^.

Bacterial and fungal metabolism of the woody substrates can lead to the production of a pool of different compounds that can be utilized by beetles as an energy source. Therefore, several bacteria and fungi identified in this research could be involved in the beetles’ nutritional roles. Their presence in the larval guts could be due to the stable associates and also depend on the habitat since the substrates on which the larvae feed can be the main determinants for the gut microbial content. Although microorganisms received from the environment may in many occasions be transient associates, some specific and functionally significant interactions can be developed *de novo* in every host generation by the acquiring of stable microbes from the environment where they live. Moreover, several fungi themselves might be an important nutritional source for the cerambycid hosts.

The microbial communities within the intestine of xylophagous larvae can perform many key functions, including lignocellulose degradation, nitrogen fixation as well as metabolism of the terpenoid molecules, which are essential to surviving of insects under extreme conditions^7^. Application of the PICRUSt software to predict the bacterial functional profiles (by using 16S rRNA sequences) allowed the inference of genes that were presumptively involved in the biotransformation of lignocellulosic compounds, through enzymes such as peroxidases, endoglucanases, alpha-L-fucosidases, mannosidases, alpha-N-arabinofuranosidases, beta-galactosidases, beta-xylosidases, beta-glucosidases, in the fixation of atmospheric nitrogen, through enzymes such as nitrogenases (Fig. 6), as well as in the metabolism of the terpenoid molecules (Supplementary Fig. S8). In almost all microbial consortia, the uncertainty of the prediction as revealed by the NSTI was low, indicating fair reliability and accuracy in the reconstruction of metagenomes. This analysis predicted the enrichment of several genes in the beetle gut bacterial communities, which were potentially involved in the biodegradation of lignocellulosic biomass. Moreover, most of the predicted bacterial genes potentially related to lignocellulose degradation were enriched in the TC, AA and AS larval gut consortia (Fig. 6).

In the work presented here, several peroxidases (EC:1.11.1-) related genes, involved in the oxidization of phenolic/non-phenolic compounds and modification of lignin polymers^59^, were predicted in all communities but were more evident in the communities of CH, AS, TC and AA. In addition, gene that encodes glycolate oxidase (EC:1.1.3.15), oxidizing glycolate to glyoxylate and producing reactive oxygen species, was predicted in all consortia. Various gluco-oligosaccharide oxidases with the ability to oxidize different carbohydrates were also observed, which might be involved in the lignocellulosic compounds transformation as well^60^. Regarding biotransformation of (hemi)cellulose, genes that encode endoglucanase (EC:3.2.1.4) (enzyme cleaving internal bonds in cellulose), alpha-L-fucosidase (EC:3.2.1.51), alpha-N-arabinofuranosidase (EC:3.2.1.55) ((hemi)cellulosic accessory enzymes catalyzing the hydrolysis of arabinans, arabinoxylans, alpha-l-fucosyl residues^61,62^), xylan 1,4-beta-xylosidase (EC:3.2.1.37) (glycosidase hydrolyzing linkage between beta-linked xylose residues in beta-1,4 xylan^63^), beta-galactosidase (EC:3.2.1.23) (enzyme hydrolyzing beta-galactosidic bonds^64^), beta-mannosidase (EC:3.2.1.25) (enzyme hydrolyzing terminal beta-D-mannose residues in beta-D-mannosides), beta-glucosidase (EC:3.2.1.21) (enzyme hydrolyzing short chain oligosaccharides and cellobiose^21^) as well as many other genes were predicted in the gut bacterial communities of all beetle larvae (Fig. 6; Supplementary Table S5). High levels of many genes for these enzymes have also been predicted in wheat straw degrading microbial consortia^65^ and observed in the microbial community decomposing poplar wood chips^66^. In addition, many of these genes, as well as several others, were detected during metagenomic analysis of a microbial community associated with the Asian cerambycid beetle (*Anoplophora glabripennis*)^21^. Concerning nitrogen fixation, the key genes, including *nifH*, *nifD*, and *nifK* for nitrogenase component proteins (EC:1.18.6.1)^67^, were predicted in all larval gut consortia, while these genes were enriched in TC, AS and CH larvae, where they might be implicated in nitrogen-fixing processes providing nitrogen to insects^68^.

## Conclusions

In conclusion, the present study characterizes the bacterial and fungal communities associated with the gut systems of several cerambycid larvae, including *A. septentrionis*, *A. aedilis*, *C. coriaceum*, *T. campestris* and *C. herbstii*. The molecular approach used here revealed that these Cerambycidae species were mostly associated with the bacterial phyla Proteobacteria and Actinobacteria and the fungal phylum Ascomycota. However, the bacterial and fungal communities varied by beetle species and between individual organisms. Remarkably, bacterial genes that encode different enzymes involved in the lignocellulose degradation and nitrogenase component proteins were indicated by predictive metagenomes reconstruction. Thus, most of the bacterial genes potentially related to lignocellulose degradation were enriched in the *T. campestris,* *A. aedilis* and *A. septentrionis* larval gut communities, whereas bacterial genes affiliated with the nitrogen fixation were enriched in the *T. campestris*, *A. septentrionis* and *C. herbstii* larval gut communities. Several bacteria and fungi identified in this work could be involved in the nutrition of beetles. Future studies that confirm the ability of many bacteria and fungi to degrade tree components are necessary to clarify their precise impacts on cerambycid beetles’ biology.

## Materials and Methods

### Collection site description and sampling procedure

Actively feeding larvae of cerambycid beetles were collected between June and September 2016 in their natural habitat in forests near city Kazan (Republic of Tatarstan, Russian Federation). Third-stage larvae of *A. septentrionis*, *A. aedilis*, *C. coriaceum*, *T. campestris* and *C. herbstii* were isolated from dead trees or stumps using sterilized tweezers and were placed into separate plastic containers with small pieces of woods. They were transported alive to the laboratory, and larvae of each species were grouped in accordance with their morphological characteristics. Then, two representatives of each beetle species were used for their further differentiation and accurate identification by using molecular analysis (by sequencing of COI and 18S rRNA genes)^19^. Abbreviations of samples in the text, figures and tables are given in accordance with the scientific names of insects (*A. septentrionis* – AS, *A. aedilis* – AA, *C. coriaceum* – CC, *T. campestris* – TC and *C. herbstii* – CH) and the order of the individual larva (1–7).

In this work, bacteria and fungi associated with the intestine of beetle larvae (acquired by larvae from the environment) were investigated. For each beetle species, seven replicates were prepared (larvae were selected from the same environment). For each replicate, one insect was carefully chosen and its gut system (midgut and hindgut) was used for total DNA extraction. Before the intestinal tracts preparation, the surface of an individual larva was thoroughly rinsed with 70% ethanol and sterile phosphate buffer. The preparation of the larval gut systems was performed on the sterile glass slides with a pair of sterilized tweezers and sterile scalpels under sterile conditions. The remaining larval cuticles were used for the host organism DNA extraction and identification of beetle species using methods of molecular biology.

### Larvae DNA extraction, PCR amplification and beetles identification

The initial morphological identification of the cerambycid beetle larvae was confirmed by sequencing of their cytochrome *c* oxidase subunit I (COI) gene and 18S rRNA gene. Genomic DNA from cuticles was extracted and purified with a FastDNA spin kit (MP Biomedicals, USA) and a FastPrep-24 homogenizer (MP Biomedicals, USA) according to the manufacturer’s protocol. The obtained genomic DNA was quantified with a NanoDrop 2000 spectrophotometer (Thermo Fisher Scientific, USA) and stored at −20 °C until use. PCR products received with the primers LCO-1480 (5′-GGT CAA CAA ATC ATA AAG ATA TTG G-3′) and HCO-2198 (5′-TAA ACT TCA GGG TGA CCA AAA AAT CA-3′) targeting mitochondrial COI gene^19^ as well as the primers CV7F (5′-CTT AAA GGA ATT GAC GGA GGG CAC CAC C-3′) and CV7R (5′-GAT TCC TTC AGT GTA GCG CGC GTG-3′) targeting the 18S rRNA gene^19^ were purified with a QIAquick PCR Purification kit (Qiagen, Germany) and subsequently sequenced on an ABI 3730 DNA Analyzer (Life Technologies, USA) according to the manufacturers’ protocols. All aligned COI gene sequences were translated to amino acid sequences to check for numts. All sequences of each marker gene were aligned using MUSCLE with default settings. Neighbor-joining tree (COI dataset) was calculated with MEGA 7 based on Kimura 2-parameter (K2P) distances^69^. Each COI sequence was submitted to Barcode of Life Data System (BOLD) as a query for animal species identification, while 18S rRNA gene sequences were compared with the SILVA rRNA database. Sequencing data (COI and 18S rRNA genes) were additionally confirmed by BLAST searches in the GenBank’s database. The nucleotide sequences received in this study were submitted to the GenBank database and available under accession numbers MF776958–MF776960, MF776962–MF776964, MG905084–MG905087 (COI gene) and MF776949–MF776951, MF776953– MF776955 (18S rRNA gene).

### Microbial DNA extraction, PCR amplification and sequencing

Total DNA was extracted with a FastDNA spin kit for soil (MP Biomedicals, USA) and a FastPrep-24 homogenizer (MP Biomedicals, USA) according to the manufacturer’s protocol. Negative extraction control samples failed to produce visible amplicons and therefore were not analyzed further. The extracted DNA was quantified with the NanoDrop 2000 spectrophotometer (Thermo Fisher Scientific, USA) and stored at −20 °C until use. The obtained DNA was then used for PCR using Q5 Hot Start High-Fidelity 2X Master Mix (BioLabs, New England) and universal primers targeting the bacterial 16S rRNA gene and fungal ITS2 region. The primers Bakt_341F (5′-CCT ACG GGN GGC WGC AG-3′) and Bakt_805R (5′-GAC TAC HVG GGT ATC TAA TCC-3′) were used to amplify V3 to V4 variable regions of the bacterial 16S rRNA gene^70^. The primers ITS3_KYO2 (5′-GAT GAA GAA CGY AGY RAA-3′) and ITS4 (5′-TCC TCC GCT TAT TGA TAT GC-3′) were used to amplify the fungal ITS2 region^17^. Each sample was amplified in triplicate and prepared for sequencing as described previously^71^. Sequencing was performed with the MiSeq system (Illumina, USA) using paired-end 2 × 300 (for 16S rRNA genes) and 2 × 250 (for the fungal ITS2 region) nucleotide dual-index sequencing at Joint KFU-Riken Laboratory, Kazan Federal University (Kazan, Russia). In addition, for one larva of each insect species high-throughput sequencing of bacterial 16S rRNA gene was conducted in duplicate (two technical replicates) to ensure reproducibility. Raw sequence data were deposited in the NCBI’s sequence reads archive (PRJNA400790), and all data are available from the researchers upon request.

### Microbial data analysis

After sequencing, all reads were processed and analyzed using the QIIME package release 1.9.1^72^. Low quality and chimeric sequences as well as low abundance sequences (lower than 5 reads per an OTU) were removed from the analysis. Sequences were clustered by the open-reference OTU clustering strategy using the default settings at 97% identity threshold. The representative 16S rRNA gene sequences were assigned to taxonomy using the Greengenes database^73^ and RDP Classifier^74^, while representative ITS sequences were taxonomically assigned using the UNITE database^75^ and BLAST assigner. In case of ITS data, all sequences not belonging to fungi were removed from further analysis. Low abundance sequences (relative abundance lower than 0.01%) were also further excluded (for both datasets). Alpha diversity values were estimated at given numbers of reads (according to the sample containing the smallest set of sequences) to avoid heterogeneity. Detected OTU numbers, Shannon, Simpson, Chao 1, Fisher’s alpha indices along with phylogenetic diversity values were estimated as the indicators for alpha diversity (followed by construction of box plots to compare the means of alpha diversity values within and between species). The nonparametric two-sample *t*-test was used to compare alpha diversity values. Non-metric multidimensional scaling (NMDS) analysis was conducted on the sample-OTU matrix using the Bray–Curtis distances.

### Reconstruction of the metagenomes with the PICRUSt software

The metagenomes of bacterial communities were reconstructed with the PICRUSt software^18^. A PICRUSt-compatible OTU table was prepared in QIIME at 97% nucleotide identity by using the closed-reference OTU collection in the Greengenes database^73^. To normalize the obtained data, we used 10,000 rarefied sequences of bacterial 16S rRNA gene per sample as an input. Then we performed the normalization by 16S rRNA copies number per OTU, and the metagenomes inference was done with the normalized OTU table^18,65^. We analyzed the average number of annotated genes in each sample and selected the top known genes affiliated with the biological transformation of lignocellulosic biomass and genes affiliated with the biological nitrogen fixation. PICRUSt software was also used to calculate the nearest sequenced taxon index.

## Electronic supplementary material

Supplementary Information
