# Supplementary material for: Comparison of intestinal bacterial and fungal communities across various xylophagous beetle larvae (Coleoptera: Cerambycidae)
Source: Sci Rep. 2018 Jul 3;8:10073. doi: 10.1038/s41598-018-27342-z (PMC6030058; doi:10.1038/s41598-018-27342-z)
Supplement: Supplementary file 1 — Supplementary Information [file 41598_2018_27342_MOESM1_ESM.pdf]

Supplementary information to the article entitled:

Comparison of intestinal bacterial and fungal communities across various xylophagous beetle larvae (Coleoptera: Cerambycidae)

Waleed S. Mohammed, Elvira E. Ziganshina, Elena I. Shagimardanova, Natalia E. Gogoleva, Ayrat M. Ziganshin

Table S1. Inter- and intraspecific genetic distances of the COI barcode fragments.

**Species 1**	**Species 2**	**K2P distance**	***p*-distance**
Acanthocinus aedilis RT05 (MF776958)	*Acanthocinus aedilis* RT20 (MF776962)	0.007	0.007
Acanthocinus aedilis RT05 (MF776958)	*Acmaeops septentrionis* RT17 (MF776960)	0.194	0.170
Acanthocinus aedilis RT20 (MF776962)	*Acmaeops septentrionis* RT17 (MF776960)	0.194	0.170
Acanthocinus aedilis RT05 (MF776958)	*Acmaeops septentrionis* RT24 (MF776964)	0.188	0.165
Acanthocinus aedilis RT20 (MF776962)	*Acmaeops septentrionis* RT24 (MF776964)	0.188	0.165
Acmaeops septentrionis RT17 (MF776960)	*Acmaeops septentrionis* RT24 (MF776964)	0.017	0.017
Acanthocinus aedilis RT05 (MF776958)	*Callidium coriaceum* RT15 (MF776959)	0.223	0.192
Acanthocinus aedilis RT20 (MF776962)	*Callidium coriaceum* RT15 (MF776959)	0.227	0.194
Acmaeops septentrionis RT17 (MF776960)	*Callidium coriaceum* RT15 (MF776959)	0.242	0.206
Acmaeops septentrionis RT24 (MF776964)	*Callidium coriaceum* RT15 (MF776959)	0.229	0.197
Acanthocinus aedilis RT05 (MF776958)	*Callidium coriaceum* RT25 (MG905084)	0.223	0.192
Acanthocinus aedilis RT20 (MF776962)	*Callidium coriaceum* RT25 (MG905084)	0.227	0.194
Acmaeops septentrionis RT17 (MF776960)	*Callidium coriaceum* RT25 (MG905084)	0.242	0.206
Acmaeops septentrionis RT24 (MF776964)	*Callidium coriaceum* RT25 (MG905084)	0.229	0.197
Callidium coriaceum RT15 (MF776959)	*Callidium coriaceum* RT25 (MG905084)	0.000	0.000
Acanthocinus aedilis RT05 (MF776958)	*Trichoferus campestris* RT21 (MF776963)	0.226	0.194
Acanthocinus aedilis RT20 (MF776962)	*Trichoferus campestris* RT21 (MF776963)	0.226	0.194
Acmaeops septentrionis RT17 (MF776960)	*Trichoferus campestris* RT21 (MF776963)	0.246	0.209
Acmaeops septentrionis RT24 (MF776964)	*Trichoferus campestris* RT21 (MF776963)	0.249	0.211
Callidium coriaceum RT15 (MF776959)	*Trichoferus campestris* RT21 (MF776963)	0.236	0.201
Callidium coriaceum RT25 (MG905084)	*Trichoferus campestris* RT21 (MF776963)	0.236	0.201
Acanthocinus aedilis RT05 (MF776958)	*Trichoferus campestris* RT26 (MG905085)	0.229	0.197
Acanthocinus aedilis RT20 (MF776962)	*Trichoferus campestris* RT26 (MG905085)	0.236	0.201
Acmaeops septentrionis RT17 (MF776960)	*Trichoferus campestris* RT26 (MG905085)	0.257	0.216
Acmaeops septentrionis RT24 (MF776964)	*Trichoferus campestris* RT26 (MG905085)	0.260	0.218
Callidium coriaceum RT15 (MF776959)	*Trichoferus campestris* RT26 (MG905085)	0.233	0.199
Callidium coriaceum RT25 (MG905084)	*Trichoferus campestris* RT26 (MG905085)	0.233	0.199
Trichoferus campestris RT21 (MF776963)	*Trichoferus campestris* RT26 (MG905085)	0.017	0.017
Acanthocinus aedilis RT05 (MF776958)	*Chlorophorus herbstii* RT27 (MG905086)	0.223	0.192
Acanthocinus aedilis RT20 (MF776962)	*Chlorophorus herbstii* RT27 (MG905086)	0.220	0.189

Acmaeops septentrionis RT17 (MF776960)	*Chlorophorus herbstii* RT27 (MG905086)	0.226	0.194
Acmaeops septentrionis RT24 (MF776964)	*Chlorophorus herbstii* RT27 (MG905086)	0.216	0.187
Callidium coriaceum RT15 (MF776959)	*Chlorophorus herbstii* RT27 (MG905086)	0.194	0.170
Callidium coriaceum RT25 (MG905084)	*Chlorophorus herbstii* RT27 (MG905086)	0.194	0.170
Trichoferus campestris RT21 (MF776963)	*Chlorophorus herbstii* RT27 (MG905086)	0.207	0.180
Trichoferus campestris RT26 (MG905085)	*Chlorophorus herbstii* RT27 (MG905086)	0.217	0.187
Acanthocinus aedilis RT05 (MF776958)	*Chlorophorus herbstii* RT28 (MG905087)	0.217	0.187
Acanthocinus aedilis RT20 (MF776962)	*Chlorophorus herbstii* RT28 (MG905087)	0.213	0.184
Acmaeops septentrionis RT17 (MF776960)	*Chlorophorus herbstii* RT28 (MG905087)	0.219	0.189
Acmaeops septentrionis RT24 (MF776964)	*Chlorophorus herbstii* RT28 (MG905087)	0.210	0.182
Callidium coriaceum RT15 (MF776959)	*Chlorophorus herbstii* RT28 (MG905087)	0.200	0.175
Callidium coriaceum RT25 (MG905084)	*Chlorophorus herbstii* RT28 (MG905087)	0.200	0.175
Trichoferus campestris RT21 (MF776963)	*Chlorophorus herbstii* RT28 (MG905087)	0.204	0.177
Trichoferus campestris RT26 (MG905085)	*Chlorophorus herbstii* RT28 (MG905087)	0.214	0.184
Chlorophorus herbstii RT27 (MG905086)	*Chlorophorus herbstii* RT28 (MG905087)	0.025	0.024

Table S2. Summary statistics for the Illumina MiSeq runs for all samples.

Sample ID	16S rRNA gene			ITS region		
	Median length	Number of received reads	Number of filtered reads	Median length	Number of received reads	Number of filtered reads
**AS_1**	445	90029	81004 (90%)	277	127894	122448 (96%)
**AS_2**	445	51765	33310 (64%)	379	298431	136666 (46%)
**AS_3**	465	175876	102184 (58%)	277	31581	30450 (96%)
**AS_4**	445	168357	115137 (68%)	277	35953	34755 (97%)
**AS_5**	447	169804	142229 (84%)	370	24652	19762 (80%)
**AS_6**	465	157732	114271 (72%)	277	57241	56074 (98%)
**AS_7**	465	153940	115981 (75%)	378	51490	45831 (89%)
**AA_1**	465	109106	85566 (78%)	346	34042	26603 (78%)
**AA_2**	442	67519	53887 (80%)	251	145708	123498 (85%)
**AA_3**	445	151134	115648 (77%)	374	44508	38598 (87%)
**AA_4**	465	128039	105460 (82%)	46	4976	11 (0%)
**AA_5**	465	127004	110826 (87%)	277	61909	59828 (97%)
**AA_6**	465	149486	135817 (91%)	368	70092	53928 (77%)
**AA_7**	465	148222	136948 (92%)	250	127611	125410 (98%)
**CC_1**	440	123236	105222 (85%)	368	67425	56651 (84%)
**CC_2**	440	140109	92099 (66%)	357	18010	11173 (62%)
**CC_3**	440	140670	125010 (89%)	387	13176	4238 (32%)
**CC_4**	445	154811	82470 (53%)	374	50397	42268 (84%)
**CC_5**	440	161272	147317 (91%)	365	12679	8206 (65%)
**CC_6**	451	145824	118432 (81%)	368	27336	23207 (85%)
**CC_7**	440	147420	124883 (85%)	367	15981	12003 (75%)
**TC_1**	445	162512	94354 (58%)	344	386935	364857 (94%)
**TC_2**	460	142711	89570 (63%)	343	7039	5241 (74%)
**TC_3**	460	149158	100832 (68%)	367	37372	31217 (84%)
**TC_4**	460	143082	120277 (84%)	374	39092	28465 (73%)
**TC_5**	465	132211	86166 (65%)	370	62487	56568 (91%)
**TC_6**	445	147086	121954 (83%)	370	37591	30830 (82%)
**TC_7**	465	161723	33310 (21%)	347	45793	36803 (80%)
**CH_1**	443	161199	110268 (68%)	375	11655	5130 (44%)
**CH_2**	452	105450	90146 (85%)	290	8004	3598 (45%)
**CH_3**	464	147352	127873 (87%)	355	10023	5622 (56%)
**CH_4**	465	131654	83685 (64%)	376	6871	3563 (52%)
**CH_5**	460	130591	106370 (81%)	390	6286	0 (0%)
**CH_6**	445	155017	100415 (65%)	367	11625	7170 (62%)
**CH_7**	459	144978	86376 (60%)	375	6240	2174 (35%)

Table S3. Summary of observed bacterial OTUs, Shannon entropy, Simpson, Chao 1, Fisher's alpha and phylogenetic diversity values.

Sample ID	OTUs	Shannon entropy	Simpson	Chao 1	Fisher's alpha	PD whole tree
**AS_1**	54	1.60	0.49	57.33	6.31	6.91
**AS_2**	42	1.93	0.55	45.00	4.75	5.75
**AS_3**	40	4.52	0.95	55.00	4.49	5.57
**AS_4**	33	3.99	0.92	40.50	3.62	3.84
**AS_5**	87	4.02	0.88	92.00	10.85	8.28
**AS_6**	30	4.18	0.94	31.00	3.25	4.57
**AS_7**	51	4.62	0.94	57.00	5.91	5.16
**AA_1**	59	2.76	0.74	60.88	6.97	6.84
**AA_2**	61	1.80	0.52	70.00	7.24	7.65
**AA_3**	38	3.42	0.82	40.00	4.24	4.28
**AA_4**	28	3.20	0.85	64.00	3.01	4.18
**AA_5**	34	2.81	0.79	35.00	3.74	4.14
**AA_6**	50	3.32	0.83	71.00	5.78	5.31
**AA_7**	65	1.45	0.55	74.00	7.78	6.56
**CC_1**	66	2.55	0.57	73.50	7.92	6.67
**CC_2**	91	3.26	0.69	92.50	11.42	8.59
**CC_3**	76	2.81	0.67	77.00	9.30	6.88
**CC_4**	110	3.97	0.79	110.08	14.19	10.12
**CC_5**	81	0.92	0.18	86.14	10.00	7.80
**CC_6**	80	4.47	0.92	84.67	9.86	8.41
**CC_7**	85	3.50	0.78	87.00	10.56	7.79
**TC_1**	94	3.81	0.84	96.00	11.85	8.58
**TC_2**	97	3.67	0.84	103.11	12.28	9.18
**TC_3**	81	5.15	0.96	82.50	10.00	7.96
**TC_4**	98	4.85	0.95	104.11	12.43	10.09
**TC_5**	62	1.80	0.57	75.33	7.38	5.47
**TC_6**	109	5.35	0.96	111.63	14.04	9.98
**TC_7**	84	3.86	0.87	88.67	10.42	8.14
**CH_1**	28	3.73	0.91	31.33	3.01	4.38
**CH_2**	20	3.44	0.89	27.50	2.07	4.12
**CH_3**	30	3.56	0.90	69.00	3.25	4.56
**CH_4**	27	3.64	0.89	42.00	2.89	4.26
**CH_5**	26	3.45	0.90	71.00	2.77	4.13
**CH_6**	33	3.31	0.79	36.33	3.62	4.42
**CH_7**	33	3.92	0.91	47.00	3.62	4.65

Alpha diversity was calculated at given number of reads 33,000.

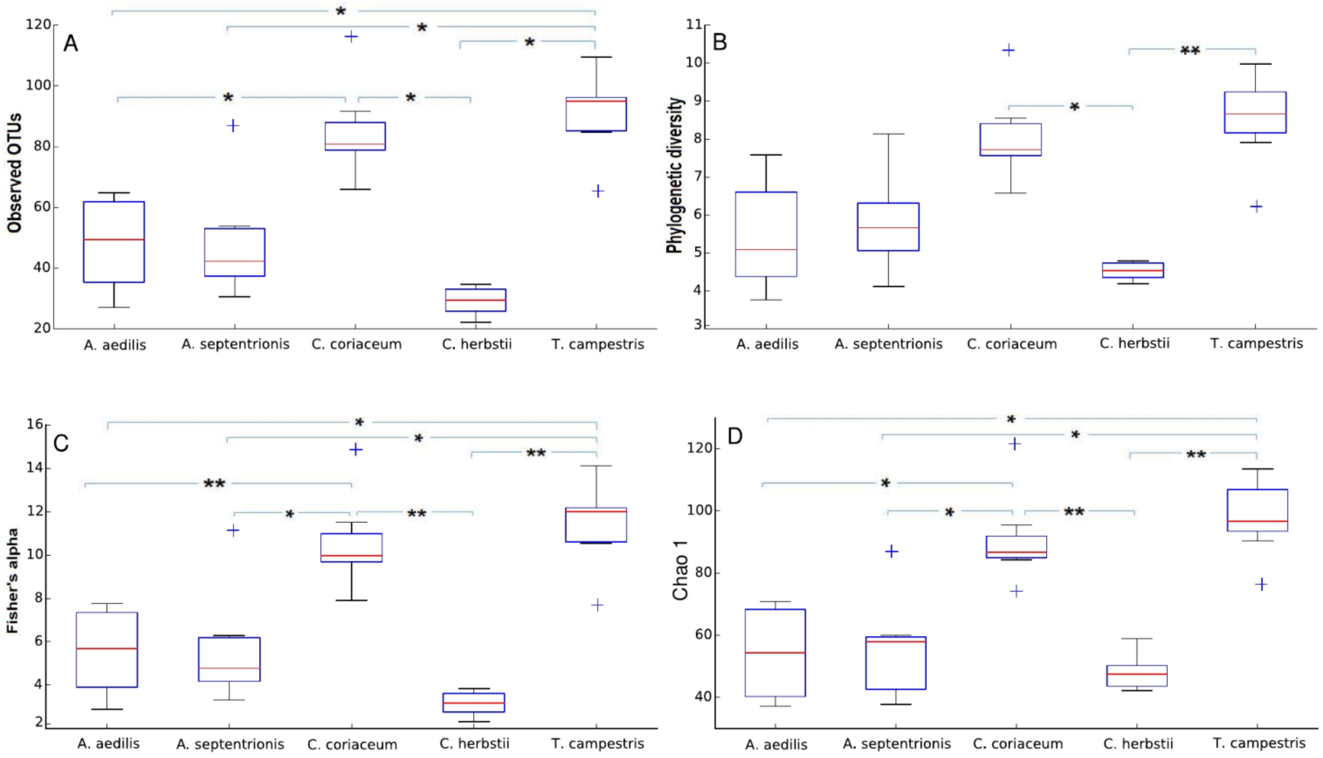

Figure S1. Box plots of observed bacterial OTUs (A), phylogenetic diversity (B), Fisher's alpha (C) and Chao 1 (D) values for comparison of bacterial diversity in larval gut samples (n=7). P values were calculated by two-sample *t*-test (*P < 0.05, **P < 0.01).

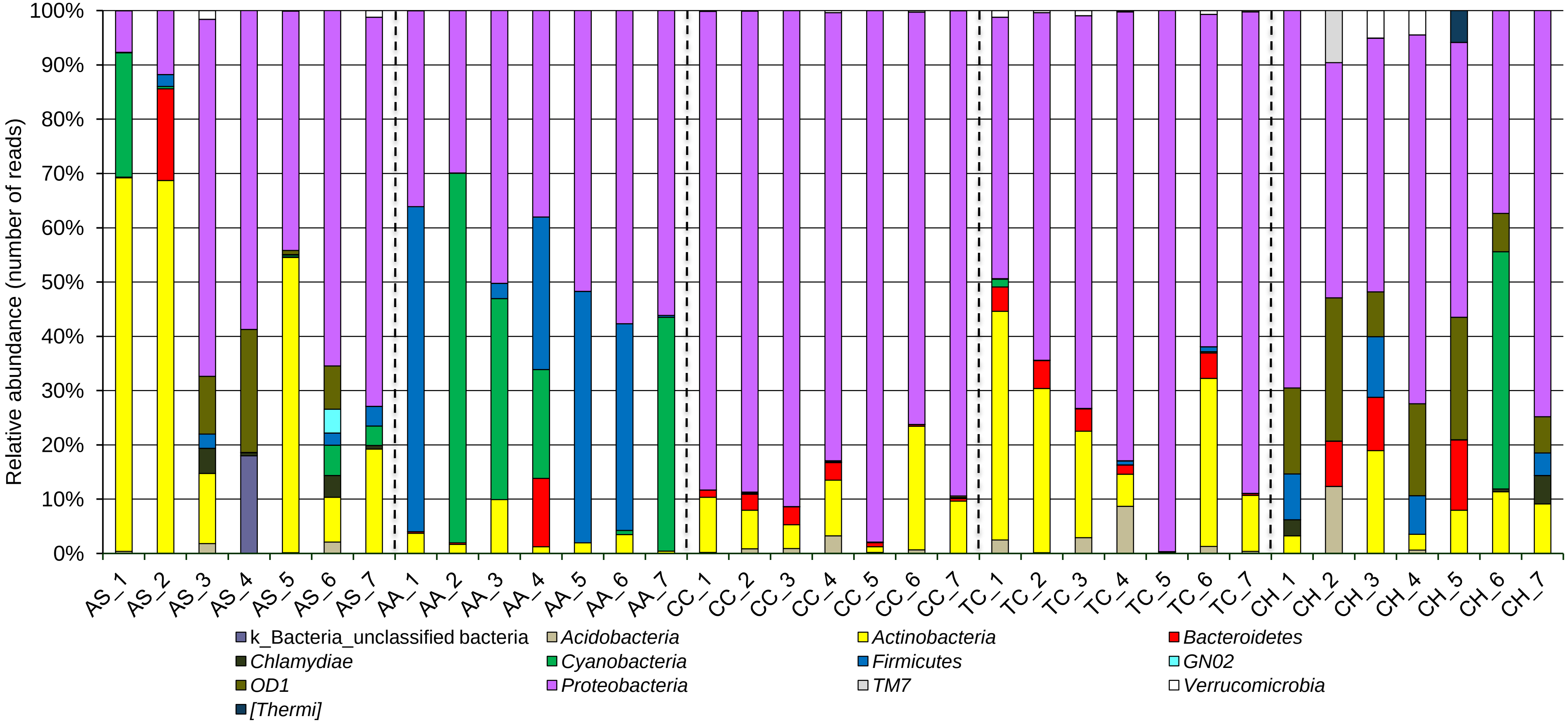

Figure S2. Relative abundance of bacterial phyla (based on 16S rRNA gene) within cerambycid larval gut samples. Abbreviations of samples in the figure are given in accordance with the scientific names of insects (*A. septentrionis* – AS, *A. aedilis* – AA, *C. coriaceum* – CC, *T. campestris* – TC and *C. herbstii* – CH) and the order of the investigated individual larva.

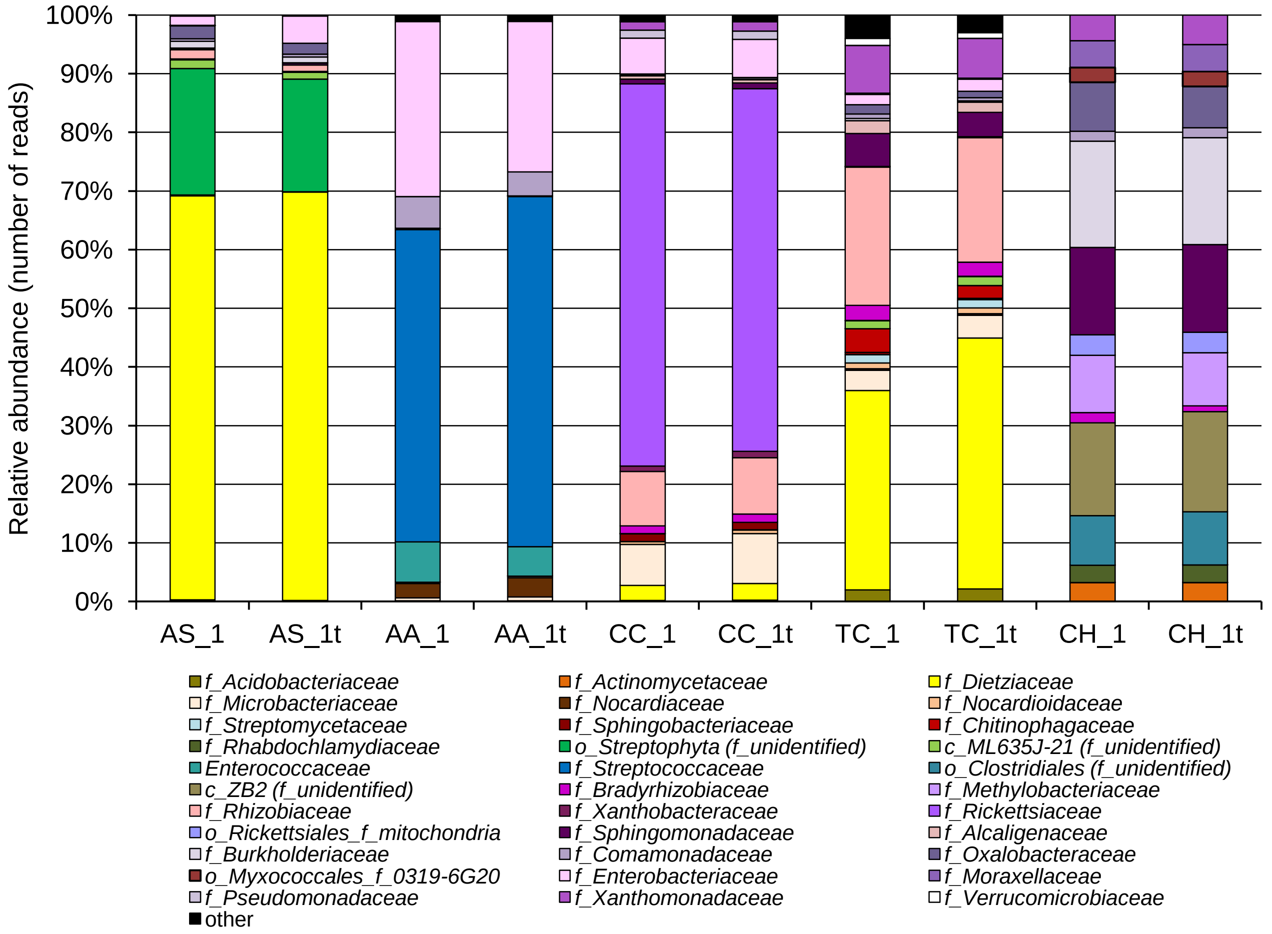

Figure S3. Relative abundance of bacterial families (based on 16S rRNA gene) within cerambycid larval gut samples (technical replicates; t). Abbreviations of samples in the figure are given in accordance with the scientific names of insects (*A. septentrionis* – AS, *A. aedilis* – AA, *C. coriaceum* – CC, *T. campestris* – TC and *C. herbstii* – CH) and the number of investigated individual larva. Only families comprising at least 1% relative abundance in at least one sample are shown. Technical replicates obtained from five samples indicate a good reproducibility of the Illumina sequencing approach.

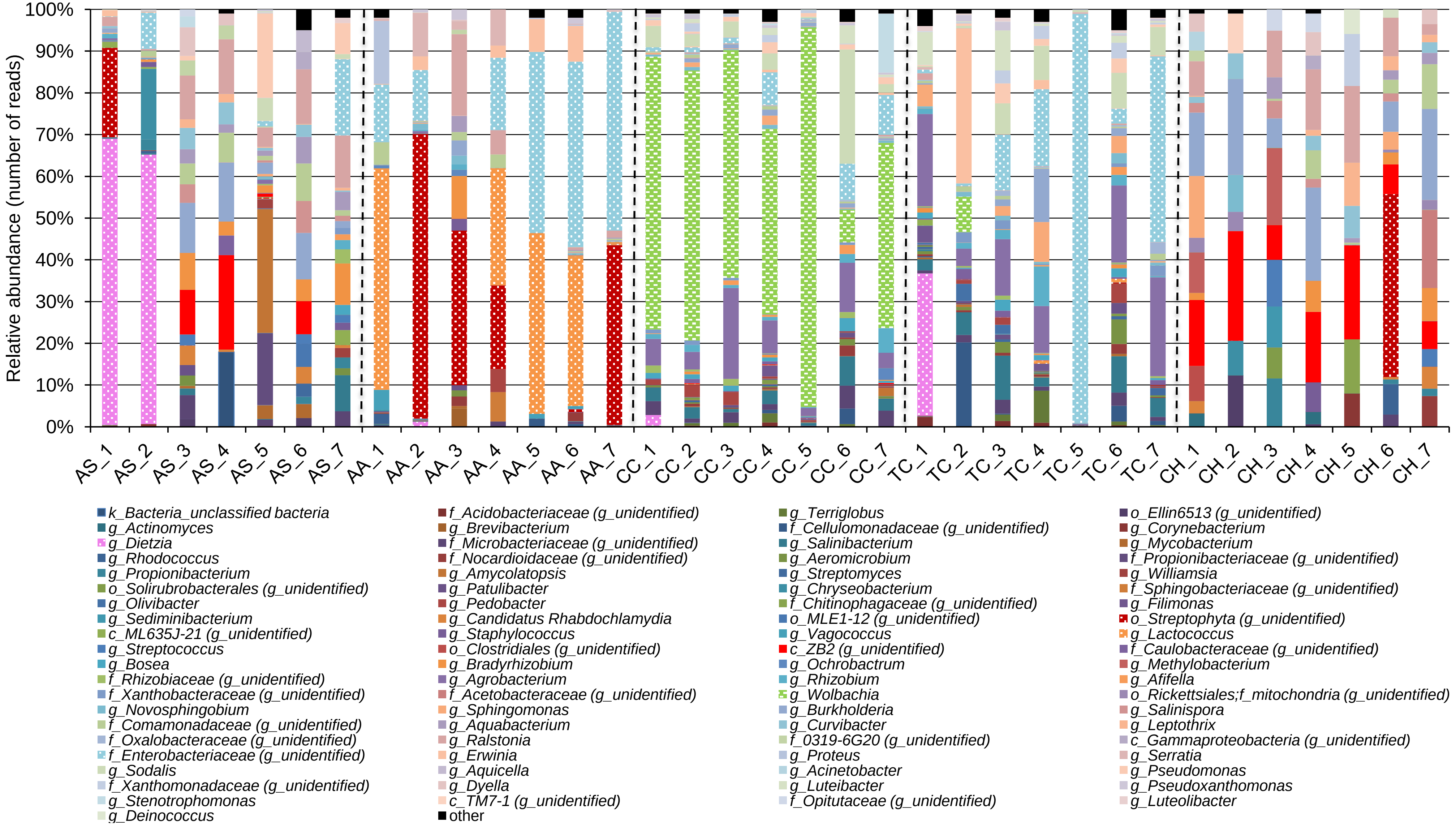

Figure S4. Relative abundance of bacterial taxa (based on 16S rRNA gene) within cerambycid larval gut samples (genus level). Abbreviations of samples in the figure are given in accordance with the scientific names of insects (*A. septentrionis* – AS, *A. aedilis* – AA, *C. coriaceum* – CC, *T. campestris* – TC and *C. herbstii* – CH) and the order of the investigated individual larva. Only taxa comprising at least 2% relative abundance in at least one sample are shown.

Table S4. Summary of observed fungal OTUs, Shannon entropy, Simpson, Chao 1 and Fisher's alpha values.

Sample ID	OTUs	Shannon entropy	Simpson	Chao 1	Fisher's alpha
**AS_1**	24	1.11	0.27	25.00	3.80
**AS_2**	3	1.08	0.51	3.00	0.34
**AS_3**	2	0.11	0.03	2.00	0.22
**AS_4**	1	0.00	0.00	1.00	0.10
**AS_5**	15	2.85	0.79	15.00	2.18
**AS_6**	4	0.09	0.02	4.00	0.48
**AS_7**	10	2.90	0.84	10.00	1.36
**AA_1**	22	2.58	0.72	22.33	3.43
**AA_2**	20	2.60	0.74	23.00	3.06
**AA_3**	7	1.25	0.48	7.00	0.90
**AA_5**	6	0.50	0.13	6.00	0.76
**AA_6**	6	2.24	0.77	6.00	0.76
**AA_7**	13	1.25	0.38	28.00	1.85
**CC_1**	38	4.03	0.91	40.50	6.59
**CC_2**	26	3.67	0.87	26.50	4.18
**CC_3**	5	1.93	0.67	5.00	0.61
**CC_4**	46	3.89	0.91	49.11	8.31
**CC_5**	12	3.05	0.86	12.00	1.68
**CC_6**	42	4.30	0.94	44.50	7.44
**CC_7**	8	2.64	0.80	8.00	1.05
**TC_1**	36	3.88	0.87	36.00	6.17
**TC_2**	46	3.85	0.85	46.60	8.31
**TC_3**	39	3.95	0.91	54.00	6.80
**TC_4**	54	4.44	0.93	54.38	10.11
**TC_5**	34	1.68	0.44	43.00	5.76
**TC_6**	54	4.17	0.92	57.00	10.11
**TC_7**	28	3.43	0.85	28.00	4.57
**CH_1**	1	0.00	0.00	1.00	0.10
**CH_2**	1	0.00	0.00	1.00	0.10
**CH_3**	3	1.34	0.57	3.00	0.34
**CH_4**	2	0.01	0.00	2.00	0.22
**CH_6**	5	1.56	0.65	6.00	0.61
**CH_7**	4	0.02	0.00	7.00	0.48

Alpha diversity was calculated at given number of reads 2,100. AA_4 and CH_5 samples were removed from the analysis due to low sequence numbers.

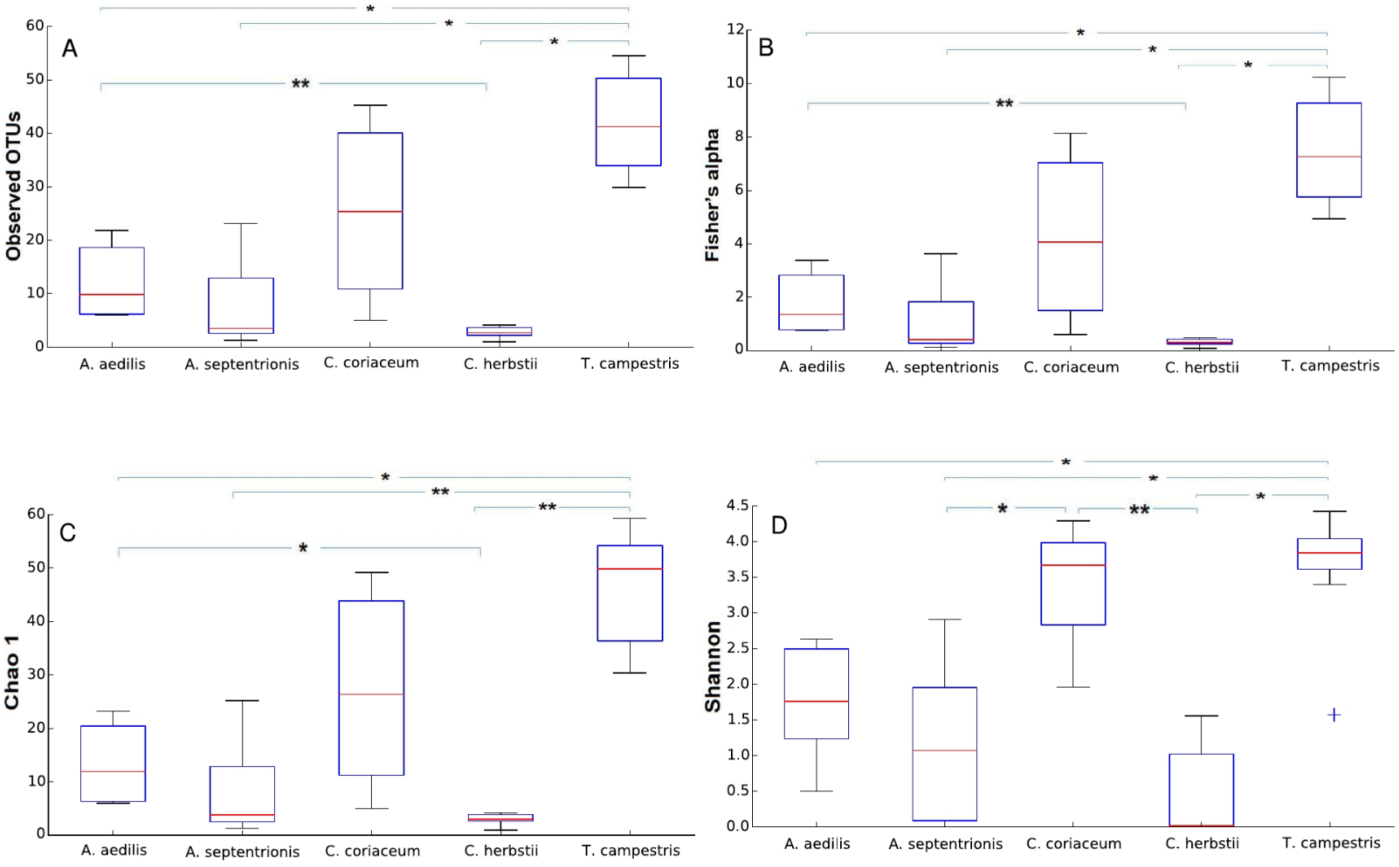

Figure S5. Box plots of observed fungal OTUs (A), Fisher's alpha (B), Chao 1 (C) and Shannon (D) values for comparison of fungal diversity in larval gut samples (*A. septentrionis*, *C. coriaceum*, *T. campestris*, n=7; *A. aedilis*, *C. herbstii*, n=6*).* P values were calculated by two-sample *t*-test (*P < 0.05, **P < 0.01).

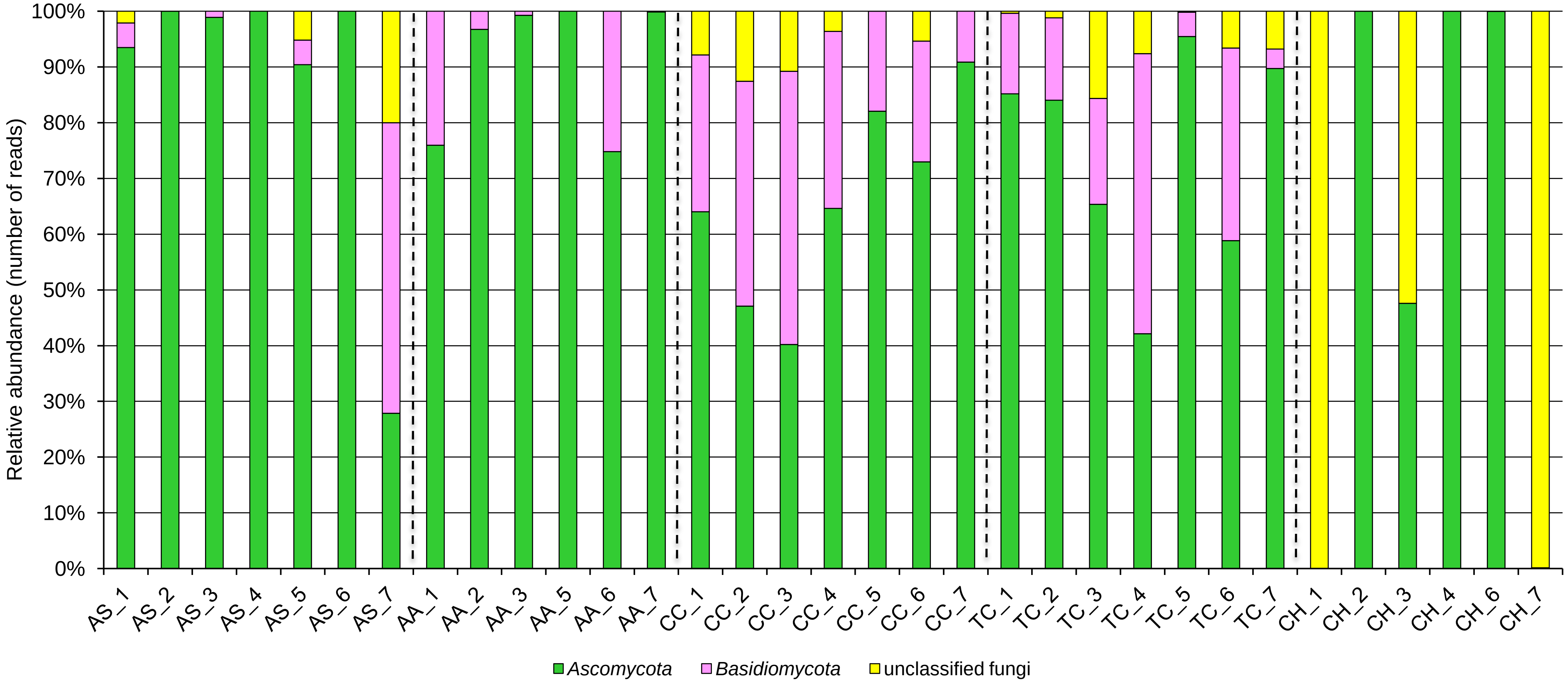

Figure S6. Relative abundance of fungal phyla (based on ITS region) within cerambycid larval gut samples. Abbreviations of samples in the figure are given in accordance with the scientific names of insects (*A. septentrionis* – AS, *A. aedilis* – AA, *C. coriaceum* – CC, *T. campestris* – TC and *C. herbstii* – CH) and the number of investigated individual larva (AA_4 and CH_5 samples were removed from the analysis due to low sequence numbers).

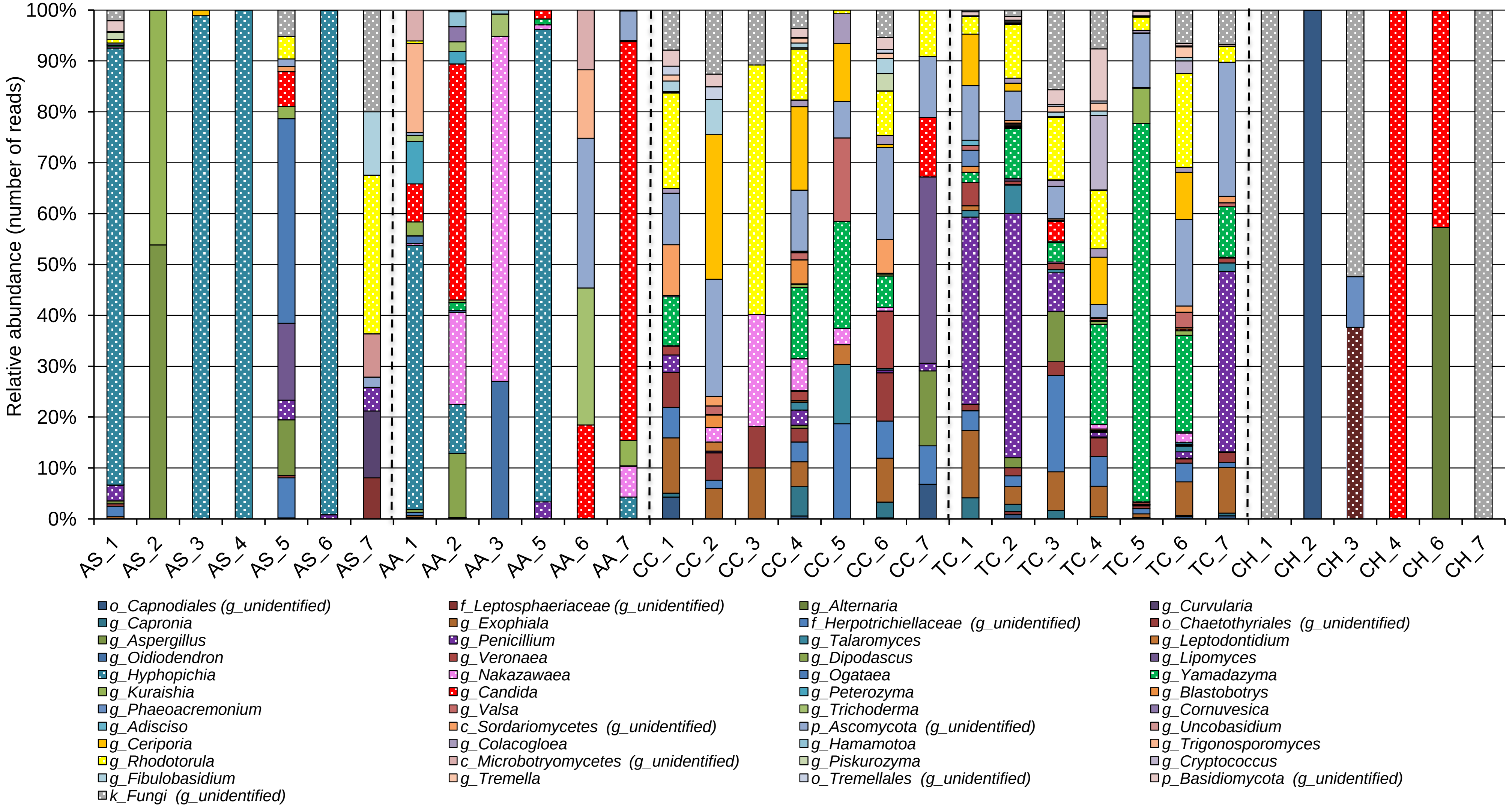

Figure S7. Relative abundance of fungal taxa (based on ITS region) within cerambycid larval gut samples (genus level). Abbreviations of samples in the figure are given in accordance with the scientific names of insects (*A. septentrionis* – AS, *A. aedilis* – AA, *C. coriaceum* – CC, *T. campestris* – TC and *C. herbstii* – CH) and the number of investigated individual larva (AA_4 and CH_5 samples were removed from the analysis due to low sequence numbers).

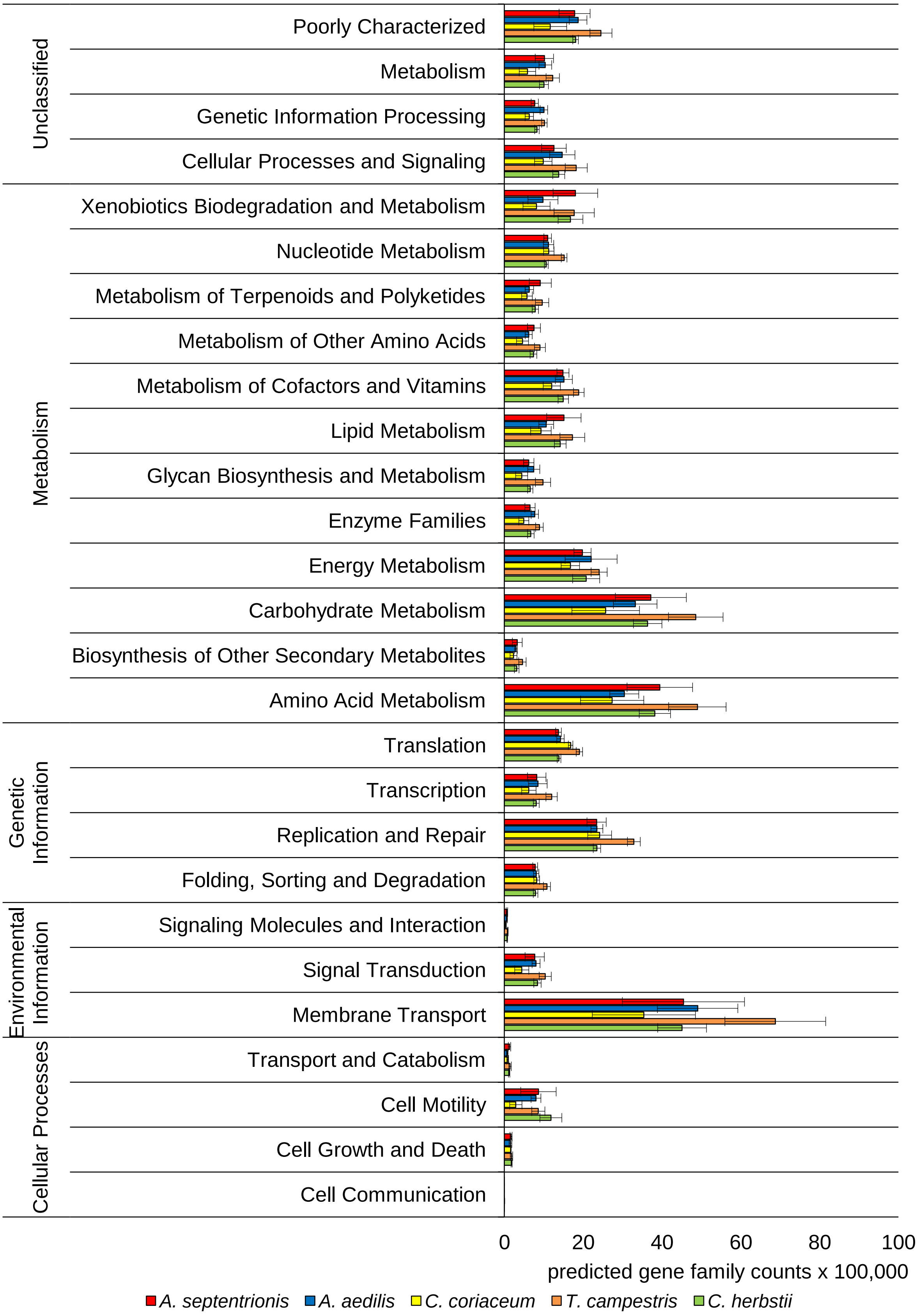

Figure S8. Comparison of predicted metabolic functions in the gut bacterial communities associated with various cerambycid larvae (mean values ± standard deviations are presented; n=7). The bacterial communities' metagenomes were reconstructed with the PICRUSt approach.

Table S5. Selection of bacterial genes involved in lignocellulose metabolism and nitrogen fixation (with the PICRUSt software) as well as the enzyme-catalyzed reactions.

	**KEGG gene description [EC number]**	**Reaction (IUBMB)**
**Lignin**	glycolate oxidase [EC:1.1.3.15]	an (S)-2-hydroxy carboxylate + O_2 = a 2-oxo carboxylate + H_2O_2
	catalase [EC:1.11.1.6]	2 H_2O_2 = O_2 + 2 H_2O
	vanillate monooxygenase [EC:1.14.13.82]	vanillate + O_2 + NADH + H^+ = 3,4-dihydroxybenzoate + NAD^+ + H_2O + formaldehyde
	catalase/peroxidase [1.11.1.21]	(1) donor + H_2O_2 = oxidized donor + 2 H_2O; (2) 2 H_2O_2 = O_2 + 2 H_2O
	glutathione peroxidase [EC:1.11.1.9]	2 glutathione + H_2O_2 = glutathione disulfide + 2 H_2O
	chloride peroxidase [EC:1.11.1.10]	RH + chloride + H_2O_2 = RCl + 2 H_2O
	cytochrome c peroxidase [EC:1.11.1.5]	2 ferrocytochrome c + H_2O_2 = 2 ferricytochrome c + 2 H_2O
**Cellulose and hemicellulose**	alpha-galactosidase [EC:3.2.1.22]	hydrolysis of terminal, non-reducing alpha-D-galactose residues in alpha-D-galactosides, including galactose oligosaccharides, galactomannans and galactolipids
	alpha-L-fucosidase [EC:3.2.1.51]	an alpha-L-fucoside + H_2O = L-fucose + an alcohol
	alpha-mannosidase [EC:3.2.1.24]	hydrolysis of terminal, non-reducing alpha-D-mannose residues in alpha-D-mannosides
	alpha-N-arabinofuranosidase [EC:3.2.1.55]	hydrolysis of terminal non-reducing alpha-L-arabinofuranoside residues in alpha-L-arabinosides
	arabinogalactan endo-1,4-beta-galactosidase [EC:3.2.1.89]	the enzyme specifically hydrolyses (1->4)-beta-D-galactosidic linkages in type I arabinogalactans
	beta-galactosidase [EC:3.2.1.23]	hydrolysis of terminal non-reducing beta-D-galactose residues in beta-D-galactosides
	beta-glucuronidase [EC:3.2.1.31]	a beta-D-glucuronoside + H_2O = D-glucuronate + an alcohol
	beta-mannosidase [EC:3.2.1.25]	hydrolysis of terminal, non-reducing beta-D-mannose residues in beta-D-mannosides
	carboxylesterase [EC:3.1.1.1]	a carboxylic ester + H_2O = an alcohol + a carboxylate
	endo-1,4-beta-xylanase [EC:3.2.1.8]	endohydrolysis of (1->4)-beta-D-xylosidic linkages in xylans
	endoglucanase [EC:3.2.1.4]	endohydrolysis of (1->4)-beta-D-glucosidic linkages in cellulose, lichenin and cereal beta-D-glucans
	levanase [EC:3.2.1.65]	random hydrolysis of (2->6)-beta-D-fructofuranosidic linkages in (2->6)-beta-D-fructans (levans) containing more than 3 fructose units

	mannan endo-1,4-beta-mannosidase [EC:3.2.1.78]	random hydrolysis of (1->4)-beta-D-mannosidic linkages in mannans, galactomannans and glucomannans
	xylan 1,4-beta-xylosidase [EC:3.2.1.37]	hydrolysis of (1->4)-beta-D-xylans, to remove successive D-xylose residues from the non-reducing termini
	6-phospho-beta-glucosidase [EC 3.2.1.86]	6-phospho-beta-D-glucosyl-(1->4)-D-glucose + H_2O = D-glucose + D-glucose 6-phosphate
	beta-glucosidase [EC:3.2.1.21]	hydrolysis of terminal, non-reducing beta-D-glucosyl residues with release of beta-D-glucose
	glucan endo-1,3-beta-D-glucosidase [EC:3.2.1.39]	hydrolysis of (1->3)-beta-D-glucosidic linkages in (1->3)-beta-D-glucans
**N_2-fixation**	nitrogenase molybdenum-iron protein alpha chain *nifD* [EC:1.18.6.1]	8 reduced ferredoxin + 8 H^+ + N_2 + 16 ATP + 16 H_2O = 8 oxidized ferredoxin + H_2 + 2 NH_3 + 16 ADP + 16 phosphate
	nitrogenase iron protein *nifH* [EC:1.18.6.1]	
	nitrogenase molybdenum-iron protein beta chain *nifK* [EC:1.18.6.1]	

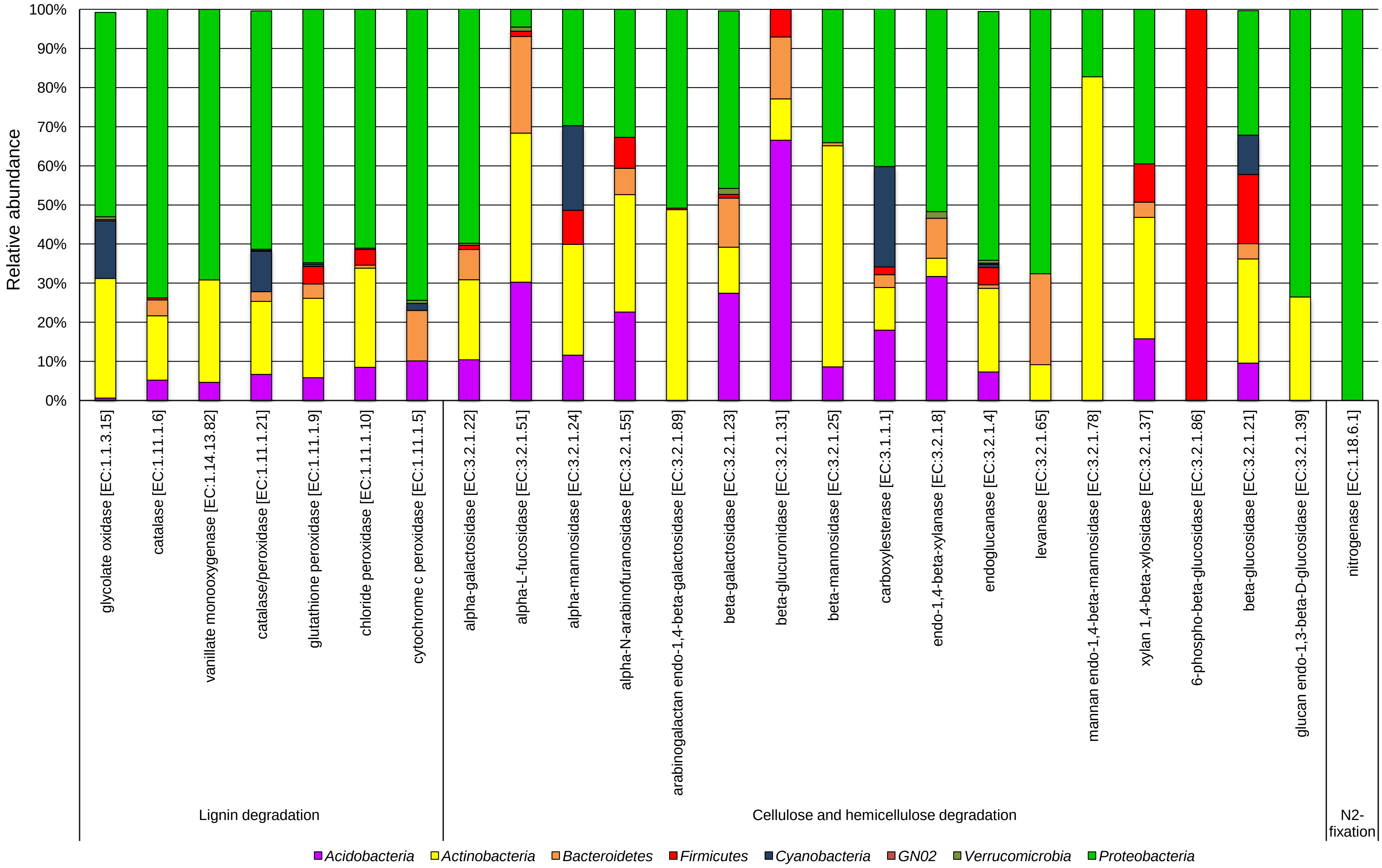

Figure S9. Bacterial phyla and their predicted functional roles (lignocellulose degradation and nitrogen fixation) identified by the PICRUSt software.
